# The Retinoblastoma Tumor Suppressor Is Required for the NUP98-HOXA9-Induced Aberrant Nuclear Envelope Phenotype

**DOI:** 10.3390/cells10112851

**Published:** 2021-10-22

**Authors:** Marcela Vaz, Birthe Fahrenkrog

**Affiliations:** 1 Laboratory Biologie du Noyau, Institute of Molecular Biology and Medicine, Université Libre de Bruxelles, 6041 Charleroi, Belgium; marcela.barros.vaz@ulb.be; 2 Biozentrum, University of Basel, 4056 Basel, Switzerland

**Keywords:** NUP98-HOXA9, retinoblastoma, nuclear envelope, PcG, histones

## Abstract

Chromosomal translocations involving the nucleoporin *NUP98* gene are recurrently identified in leukemia; yet, the cellular defects accompanying NUP98 fusion proteins are poorly characterized. NUP98 fusions cause changes in nuclear and nuclear envelope (NE) organization, in particular, in the nuclear lamina and the lamina associated polypeptide 2α (LAP2α), a regulator of the tumor suppressor retinoblastoma protein (RB). We demonstrate that, for NUP98-HOXA9 (NHA9), the best-studied NUP98 fusion protein, its effect(s) on nuclear architecture largely depend(s) on RB. Morphological alterations caused by the expression of NHA9 are largely diminished in the absence of RB, both in human cells expressing the human papillomavirus 16 E7 protein and in mouse embryonic fibroblasts lacking RB. We further show that NHA9 expression associates with distinct histone modification. Moreover, the pattern of trimethylation of histone H3 lysine-27 is affected by NHA9, again in an RB-dependent manner. Our results pinpoint to an unexpected interplay between NUP98 fusion proteins and RB, which may contribute to leukemogenesis.

## 1. Introduction

Chromosomal translocations affecting the *NUP98* gene are recurrently identified in hematopoietic malignancies. These rearrangements result in fusion proteins involving the N terminus of NUP98 (N98) fused in frame to about 30 distinct partner proteins [1,2,3,4]. The fusion partners include various homeodomain (HD) transcription factors as well as other chromatin-binding proteins. N98 is an integral part of the nuclear pore complex (NPC), and it is roughly comprised of two domains: an N-terminal GLFG (glycine–lysine–phenylalanine–glycine) repeat domain, which serves as a docking site for nuclear transport factors, and a C-terminal auto-proteolytic domain [5]. N98 is critical for selective nucleocytoplasmic transport [6,7,8,9], the barrier function of NPCs [6,7], and has been attributed several regulatory roles in gene expression: N98 is recruited to actively transcribed genes in *Drosophila* and mammalian cells [10,11], including developmental genes [12,13]; it promotes transcriptional memory for interferon-γ-inducible genes [14]; and participates in the regulation of immune response genes upon viral infections [15]. During mitosis, N98 administers mitotic spindle assembly [16] and is a temporal regulator of the anaphase-promoting complex [17,18].

N98 fusion proteins are collectively localized to the nucleus [19,20,21,22,23]. The impact of N98 fusion proteins on transcriptional targets has been studied extensively [20,22,24,25,26,27], while their potential impact on cellular function has received minor attention. We have previously shown that N98 fusion proteins collectively provoke morphological alterations in the nuclear envelope (NE), despite their heterogeneous composition and intranuclear localization [23]. These alterations in the NE consistently occurred in transiently transfected human cell lines, in transduced mouse bone marrow cells, as well as in patient derived samples. The changes correlated with modifications in the nuclear lamina and the lamina-associated polypeptide 2α (LAP2α). Concordantly, a recent proteomic screen unraveled LAP2α as a common interactor of five distinct N98 fusion proteins [24]. LAP2α is a direct binding partner of the lamina constituents lamin A/C (LA/C), and the LA/C-LAP2α complex is an important regulator of the tumor suppressor retinoblastoma protein (RB; [28,29,30]). RB and the RB-related proteins p107 and p130, known as the “pocket proteins”, are transcriptional repressors of genes involved in cell cycle progression because of their interaction with the E2 factor (E2F) family of transcription factors [28]. The pocket proteins have potentially overlapping functions and are expressed at different time points during the cell cycle and are likely to have different, but overlapping, expression patterns in vivo [31,32,33]. The LA/C–LAP2α complex primarily binds to pocket C of hypophosphorylated RB, which prevents E2F-dependent transcription and cell cycle progression [28,29,30,34]. While regulation of E2F transcription factors reflects the canonical function of RB, recent research has disclosed several noncanonical RB-dependent pathways, such as DNA repair, telomere maintenance, and epigenetic silencing of repetitive DNA sequences (reviewed in: [30,31,32]). In this context, RB has long been known to interact with chromatin regulators, such as histone deacetylates and demethylases [35,36]. A-type lamins in concert with LAP2α likewise bind chromatin remodeling factors, such as the high mobility group protein N5 (HMGN5), as well as heterochromatin and euchromatin directly [37,38,39,40]. Interestingly, N98, N98 fusion proteins, RB, and LAP2α associate with histone modifications and epigenetic chromatin regulation. For example, N98 and the best-studied N98 chimera NUP98–HOXA9 (NHA9), which originates from a t(7;11)(p15;p15) chromosomal translocation, interacts with MLL (mixed lineage leukemia). MLL, which is part of the trithorax complex, deposits the histone 3 lysine-4 trimethylation (H3K4me3) mark to developmentally regulated genes [4,12,41]. N98 fusion proteins consequently appear to colocalize with H3K4me3 sites [42,43,44]. RB facilitates heterochromatin formation through recruitment of histone deacetylase complexes, such as nucleosome remodeling and deacetylase (NuRD), and histone methyltransferases, such as EZH2 (enhancer of zeste homology 2), to deacetylate and trimethylated H3K27 at repetitive sequences [45,46,47]. Changes in LAP2α localization, on the other hand, caused defects in H3K9me3 and formation of constitutive heterochromatin [48].

In this study, we report that NE aberrations brought forward by expression of NHA9 do not occur in cells lacking the RB proteins. Moreover, loss of RB diminished the correlation of NHA9 with the facultative heterochromatin mark histone H3 lysine-27 trimethylation (H3K27me3) and the interface between RB and polycomb complexes, important for the deposition of the H3K27me3 mark.

## 2. Materials and Methods

All experiments were carried out at room temperature (RT) unless otherwise stated.

### 2.1. Cell Culture and Transfections

Mouse embryonic fibroblasts (MEFs) were obtained from Dr. Roland Foisner (Medical University, Vienna, Austria). MEFs were grown in Dulbecco’s modified Eagle’s medium (DMEM) supplemented with 10% fetal bovine serum (FBS; Biochrom GmbH, Berlin, Germany) and 1% penicillin/streptomycin (P/S; Thermo Fisher Scientific, Gibco, Merelbeke, Belgium).

HCT-116 cells were obtained from Dr. Denis Lafontaine (Université Libre de Bruxelles, Belgium). Stable HPV 16 E7 and E7(C24G)-expressing cells were established in HCT-116 cells. pCMV-16 E7 and pCMV-16 E7(C24G) plasmids were transfected using jetPRIME (Polyplus, Illkirch, France) and incubated for 48 h after dilution into fresh McCoy’s 5A medium containing 600 μg/mL of geneticin (Gibco). Single colonies were selected after 2 to 3 weeks, expanded as clone pools by passaging the cells on individual dishes, and tested for expression of HPV 16 E7 or E7(C24G) by Western blotting and immunofluorescence. Selected clones were stably maintained in McCoy’s 5A medium containing 600 μg/mL of geneticin. Transient transfections of GFP-N98 and GFP-NHA9 were performed using Lipofectamine 2000 (Thermo Fisher Scientific, Invitrogen, Merelbeke, Belgium) or jetPRIME, according to the instructions of the manufacturer. Cells were analyzed 48 h post-transfection.

### 2.2. Plasmids

pEGFP-N98 and pEGFP-NHA9 were produced as described previously [23]. The wildtype pCMV-16 E7 and pCMV-16 E7(C24G) constructs were a kind gift from Karl Munger (Addgene plasmid #85035 and #13692; [49]). Plasmids were sequenced to confirm the presence of corresponding mutations.

### 2.3. Antibodies

The following monoclonal antibodies for Western blotting (WB) and immunofluorescence (IF) were used in this study: mouse anti-lamin A/C (Abcam, Cambridge, UK, ab40567; IF 1:30), mouse anti-histone H3K9me3 (Active Motif, Waterloo, Belgium, 39285; IF 1:200), mouse anti-retinoblastoma (BD Biosciences, Erembodegem, Belgium, 554136; IF 1:200, WB 1:2000), mouse anti-HPV16 E7 (Santa Cruz, Heidelberg, Germany, sc6981; WB 1:200), mouse anti-β-tubulin (Millipore, Burlington, MA, USA, ab18251; WB 1:4000), rat anti-Nup98 (Sigma-Aldrich, Lyon, France, N1038; WB 1:1000), and rat anti-GFP (ChromoTek, Planegg-Martinsried, Germany, #029762; WB 1:1000). The following polyclonal antibodies were used in this study: rabbit anti-LAP2α (Abcam, ab5162; IF 1:250; WB 1:2500), rabbit anti-lamin A/C (Proteintech, Manchester, UK, 10298-1-AP, IF 1:100), rabbit anti-histone H3K4me3 (Cell Signaling, Leiden, The Netherlands, 9751; IF 1:400), rabbit anti-histone H3 (Cell Signaling, 9715; IF 1:250), rabbit-anti histone H3K27me3 (Active Motif, 39156; IF 1:400; WB 1:1000), rabbit anti-BMI-1 (Cell Signaling, D20B7; IF 1:200, WB 1:2000), rabbit anti-EZH2 (Cell Signaling, D2C9; IF 1:200, WB 1:2000), rabbit anti-SUZ12 (Cell Signaling, D39F6; IF 1:200, WB 1:2000), rabbit anti-RING1A (Cell Signaling, D2P4D; IF 1:200), and rabbit anti-actin (Sigma–Aldrich, Overijse, Belgium, A2066; WB 1:1000).

Secondary antibodies for immunofluorescence were goat anti-mouse IgG-Alexa 568 (Thermo Fisher Scientific, Invitrogen) and goat anti-rabbit IgG-Alexa 568 (Thermo Fisher Scientific, Invitrogen). All antibodies were used at a dilution of 1:1000. For Western blot, secondary goat anti-mouse, goat anti-rabbit, and goat anti-rat IgG coupled with alkaline phosphatase antibodies (Sigma-Aldrich) were used at a dilution of 1:10,000.

### 2.4. Immunofluorescence Microscopy

Cells were grown on glass coverslips in 24-well plates coated with poly-L-lysine and fixed in 4% formaldehyde in PBS for 5 min, permeabilized with 0.5% Triton-X-100 in PBS for 5 min, and then fixed again for another 5 min in 4% formaldehyde. Blocking was performed with PBS/2% BSA for 30 min. Next, cells were stained with the appropriate primary antibody for 2 h at RT or overnight at 4 °C in a humidified chamber. Next, cells were washed three times with PBS/2% BSA and incubated with secondary Alexa Fluor conjugated antibodies (Thermo Fisher Scientific, Invitrogen). Excess antibodies were removed by three 5 min washing steps in PBS. The coverslips were mounted with Mowiol-488 (Sigma-Aldrich) containing 1 μg/mL DAPI and stored at 4 °C until viewed. Images were acquired using a 63× oil immersion objective on an LSM710 laser-scanning confocal microscope (Zeiss, Oberkochen, Germany) or on a Zeiss Axio Observer Z.1 microscope.

### 2.5. Calculation of Pearson’s Coefficients

Quantification was performed with an intensity correlation coefficient-based method using an ImageJ plugin (version 2.1.0). Pearson’s correlation coefficient (PCC) was the statistical model used for quantifying correlation:

$$r_{\mathrm{xy}}=\frac{\sum_{i=1}^{n}\left(x_{i}-\bar{x}\right)(y_{i}-\bar{y})}{\sqrt{\sum_{i=1}^{n}{\left(x_{i}-\bar{x}\right)}^{2}}\sqrt{\sum_{i=1}^{n}{\left(y_{i}-\bar{y}\right)}^{2}}}$$

where $x_{i}$ and $y_{i}$ refer to the intensity values of the magenta and green channels, respectively, $\bar{x}$ and $\bar{y}$ refer to the mean intensities of the magenta and green channels, respectively, across the nucleus, and *n* represents the total number of segmented pixels in both images. The aim is to measure the relationship between the signal intensities in one region of interest and the corresponding values in another. PCC values range from +1 to −1, if there is no relationship between the proteins, the expected PCC is 0. A positive PCC means the two proteins are colocalized to some extent; a negative PCC value indicates that the distributions of the two probes are inversely related, for example, if one protein is restricted to the cell nucleus, and a second is localized in the cytoplasm [50]. Each individual cell was outlined by the ImageJ selection tool and measured at the same threshold settings. For each pair of staining, ~50 cells per staining were measured, and presented as mean ± SEM. Statistical analyses evaluating colocalization were performed with Student’s *t*-test for pair comparisons.

### 2.6. Foci Counting

For evaluations of foci/spots counting, a macro for automated quantitative analysis of foci was developed with ImageJ. The macro included an ROIs Selection based on DAPI image, Thresholding and Analyzing, followed by foci count.

### 2.7. GFP-Trap Magnetic Agarose Assays

HCT-116 cells stably expressing HPV 16 E7 and E7(C24G), respectively, grown in a 10 cm dish, were transfected with plasmids encoding GFP, GFP-N98, and GFP-NHA9, respectively. Cells were grown for 48 h at 37 °C in a humidified atmosphere with 5% CO_2_. To harvest cells, the growth medium was aspirated off, 1 mL of ice-cold PBS was added to cells, and the cells were scraped from the dish. Cell pellets were collected to a precooled tube and centrifuged at 4 °C at 500× *g* for 5 min; the supernatant was discarded. The pellet was washed twice with ice-cold PBS, resuspended in 200 μL of ice-cold lysis buffer (10 mM Tris/HCl, pH 7.5, 150 mM NaCl, 0.5 mM EDTA, 0.5% NP-40, 0.1% sodium azide, plus protease inhibitor tablets (Roche, Basel, Switzerland)) by pipetting up and down, and incubated 15 min on ice. The tubes were centrifuged at 4 °C at 16,000× *g* for 30 min; the supernatants were collected and stored at −80 °C. The magnetic beads were prewashed twice with 10 mM Tris/HCl, pH 7.5, 150 mM NaCl, 0.5 mM EDTA, 0.018% sodium azide, and protease inhibitors. For GFP-trap assays, 300 μg of protein lysate adjusted to 500 μL in dilution buffer (10 mM Tris/HCl, pH 7.5, 150 mM NaCl, 0.5 mM EDTA, protease–phosphatase inhibitor) was added to 25 μL of GFP-trap_MA beads (ChromoTek) for 1 h at 4 °C on an end-to-end rotor. Beads were prewashed twice with dilution buffer. The beads were then washed three times in dilution buffer containing 150 mM, 250 mM, and 500 mM NaCl, followed by the addition of 20 μL of 2× SDS-sample buffer (120 mM Tris/HCl, pH 6.8, 20% glycerol, 4% SDS, 0.04% bromophenol blue, 10% β-mercaptoethanol), and boiled at 95 °C for 10 min. The eluates were subsequently loaded on to polyacrylamide gels and the Western blot was carried out.

### 2.8. Western Blotting

Cells were lysed in lysis buffer (50 mM Tris-HCl, pH 7.8, 150 mM NaCl, 1% Nonidet-P40 and protease inhibitor tablets). In total, 20 µg of protein were loaded and separated by SDS-PAGE (sodium dodecyl sulphate–polyacrylamide gel electrophoresis) for 90 min at 100 V and the proteins were transferred onto a PVDF membrane (Immobilon-P, Merck Millipore, Overijse, Belgium) using the Trans-Blot Turbo Transfer System (7 min, 1.3 A constant, up to 25 V; Bio-Rad, Temse, Belgium). The membrane was incubated for 1 h in TBS containing 0.1% Tween 20 (TBS-T) and 5% of nonfat dry milk, followed by incubation of the primary antibodies in blocking solution overnight at 4 °C. After washing three times with TBS-T, the membrane was incubated with the appropriate alkaline phosphatase conjugated secondary antibody for 1 h. After three washes with TBS-T, the membrane was washed twice with assay buffer (100 mM Tris-HCl, pH 9.8, 10 mM MgCl_2_) for 2 min. The membrane was incubated for 5 min with the Lightning CDP Star Chemiluminescence reagent (ThermoFisher Scientific, Applied Biosystem) and developed. X-ray films were scanned and processed using Fiji/ImageJ, version 2.1.0.

### 2.9. Proximity Ligation Assays

Proximity ligation assays were performed according to the instructions of the manufacturer (Duolink; Olink Bioscience, Uppsala, Sweden). Cells were grown on glass coverslips coated with poly-L-lysine, fixed, and permeabilized as described above for immunofluorescence experiments. After incubation with primary antibodies, the cells were subjected to the Duolink red kit. In brief, after washing with Duolink wash buffer A, cells were incubated with the Duolink PLA probes for 1 h at 37 °C in a preheated humidified chamber. Following washing steps with Duolink wash buffer A, the Duolink ligation reagent was incubated for 30 min followed by the Duolink amplification reagent for 90 min, both at 37 °C in a preheated humidified chamber. Cells were washed with Duolink wash buffer B and mounted with the Duolink in situ mounting medium containing DAPI. Images were acquired using a 63× oil immersion objective on a Zeiss Axio Observer Z.1 fluorescence microscope, recorded with Axiovison software (version 4.8.2), and processed using Fiji/ImageJ. PLA foci were counted using a macro written on Fiji/ImageJ.

### 2.10. Statistical Analyses

All plots and statistics were generated using GraphPad Prism (Version 8; GraphPad Software Inc., San Diego, CA, USA) or Apple Numbers (Apple Inc., Cupertino, CA, USA). A two-tailed t-test was performed. During evaluation of the results a confidence interval α of 95% and *p* values lower than 0.05 were considered statistically significant.

### 2.11. Image Design

Schematic representations were designed using the open-source software Inkscape 0.91 (by running Xquartz 2.7.11 on macOS).

## 3. Results

### 3.1. NHA9 Alters the Nuclear Architecture in Mouse Embryonic Fibroblasts

N98 chimeras provoke deformation of nuclei and morphological alterations in the NE, which coincide with gross changes in the nuclear lamina, in particular a redistribution of LA/C, lamin B1 (LB1), and LAP2α [23]. As the LA/C–LAP2α complex acts as an important regulator of RB, we set out to further explore the link between the NHA9 fusion protein and RB. We first transiently expressed GFP-tagged versions of NHA9 and N98 in wildtype (WT) mouse embryonic fibroblasts (MEFs) and in triple *RB*^−/−^, *p107*^−/−^, *p130*^−/−^ knockout (TKO) MEFs. Due to the potential functional redundancy of the pocket proteins (see above), TKO MEFs achieve a complete loss of RB-like activity.

**Figure 1:**
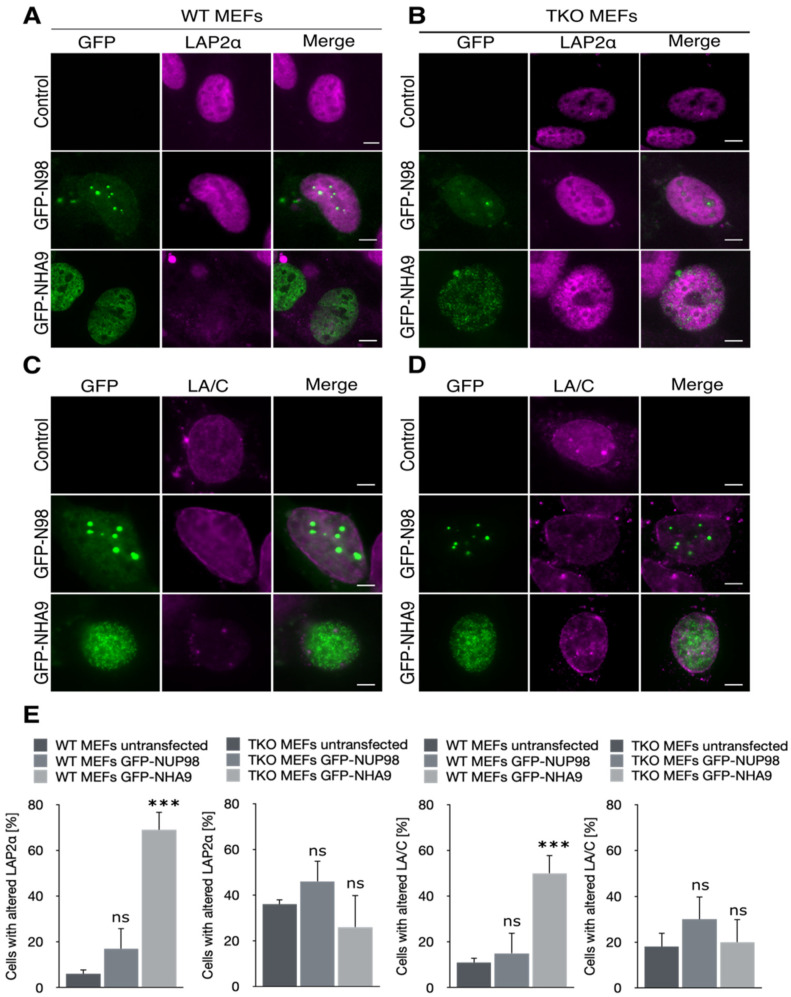
NUP98–HOXA9 perturbs LAP2α and lamin A/C distribution in MEFs. Wildtype and RB triple knockout (TKO) mouse embryonic fibroblasts (MEFs) were transiently transfected with the indicated GFP constructs, fixed, and stained for immunofluorescence microscopy 48 h post-transfection. (**A**) In MEFs expressing GFP-NUP98–HOXA (GFP-NHA9, green), LAP2α (magenta) intensity was diminished in the nucleoplasm, (**B**) while in TKO MEFs LAP2α intensity was similar to the control and GFP-NUP98 (GFP-N98, green) expressing cells. (**C**) GFP-NHA9 (green) expression in MEFs caused a reduced intensity of LA/C (magenta) at the NE, (**D**) but not in TKO MEFs. Shown are representative confocal images. Scale bars, 5 µm. (**E**) Quantification of cells with altered LAP2α and LA/C distribution. Total number of analyzed cells is indicated at the bottom of each bar. The number of experiments for LAP2α staining was: *n*  =  4; number of experiments for LA/C staining were: *n*  =  3; *** *p*  <  0.01, ns, *p* not significant, *t*-test, two-tailed, normalized to untransfected MEFs.

By direct fluorescence, we found that, in both WT and TKO MEFs, GFP-NHA9 localized to the nucleoplasm in a punctate pattern (Figure 1A) as previously described by us and others [2,4,22,51]. GFP-N98 on the other hand localized to the nuclear rim in a punctuated pattern typical for nucleoporins, to the nucleoplasm, and in nuclear foci (Figure 1A). We at the same time examined the fate of the LAP2α in the GFP-NHA9 and GFP-N98 expressing MEFs by immunofluorescence microscopy. As shown in Figure 1A,B, the LAP2α signal was significantly reduced in WT MEFs expressing GFP-NHA9, but not in TKO MEFs.

In contrast, LAP2α was homogenously distributed throughout the nucleoplasm in WT and TKO MEFs expressing GFP-N98, comparable to the untransfected control cells (Figure 1A,B). The plot profile of the fluorescence intensity revealed a reduction in LAP2α intensity in WT MEFs expressing GFP-NHA9 compared to GFP-N98 expressing cells, whereas the LAP2α intensity profile was not different in TKO MEFs expressing GFP-NHA9 or GFP-N98 (Supplementary Figure S1A). Overall, about 70% of WT MEFs expressing GFP-NHA9 exhibited reduced LAP2α intensity but less than 20% of GFP-N98 expressing cells. In contrast, only ~25% of the GFP-NHA9 expressing TKO MEFs exhibited reduced LAP2α intensity and ~45% of GFP-N98 expressing cells, alike the untransfected control cells (Figure 1E). Similarly, expression of GFP-NHA9 in WT MEFs caused a reduction in LA/C intensity at the nuclear lamina (Figure 1C and Figure S1B), which was not the case in TKO MEFs (Figure 1D,E). Note that the distribution of both LAP2α and LA/C in TKO MEFs was more frequently altered in untransfected cells, which may be due to the disruption of the RB–LAP2α–LA/C complex in these cells [28]. Despite the reduced intensity of the LAP2α fluorescence, we did not observe significant changes in the protein levels of LAP2α upon expression of GFP-NHA9 or GFP-N98 in either WT and TKO MEFs, just as for LB1 (Figure S1C). Together, our data suggest that NHA9 may induce nuclear architecture changes in a retinoblastoma-dependent manner.

### 3.2. NHA9 Provokes Changes in Nuclear Architecture Only in the Presence of the Pocket Proteins

To strengthen the notion that RB plays an important role in the genesis of nuclear architecture changes elicited by N98 fusion proteins, we next employed the human colon carcinoma cell line HCT-116 (ATCC: CCL-247). We developed stable cell lines with the respective expression of the active E7 protein of human papillomavirus (HPV) 16 and its inactive E7(C24G) form. E7 promotes the proteosomal degradation of RB, p107, and p130 [52], whereas the C24G mutant of E7 is unable to bind the RB family proteins [53]. Consistently, RB was absent in HCT-116 cells expressing E7 but not in E7(C24G) expressing cells (Figure S1D).

**Figure 2:**
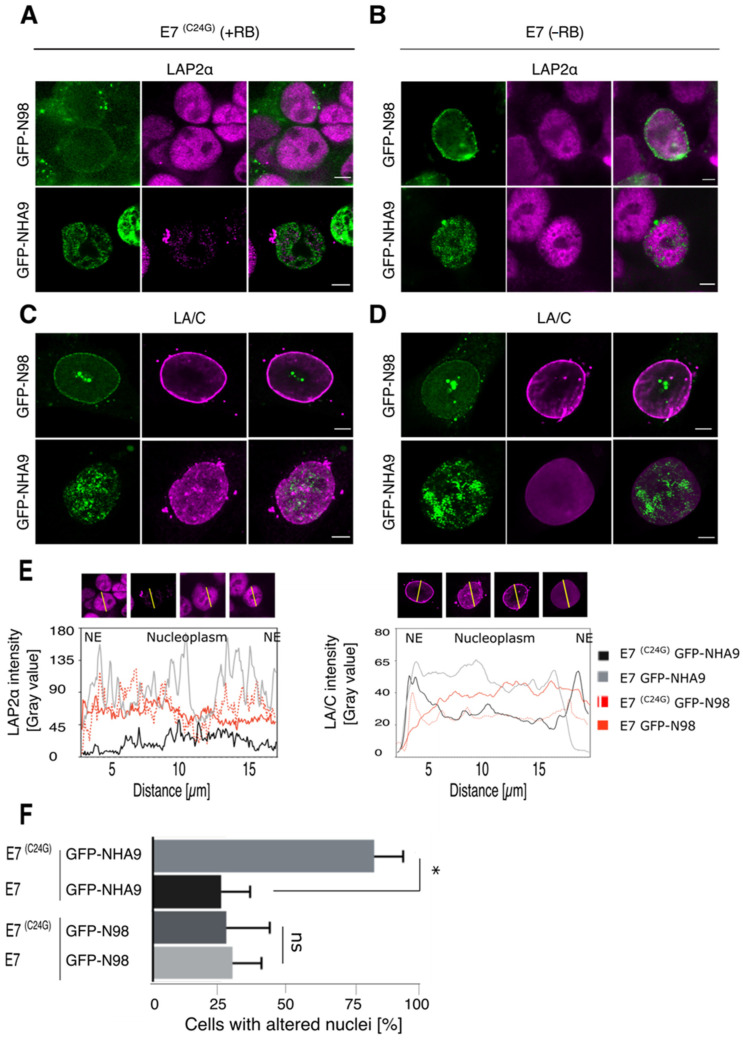
NUP98–HOXA9 affects LAP2α and lamin A/C distribution in E7(C24G) expressing cells. HCT-116 cells stably expressing the HPV16 E7 protein (E7) or its inactive variant E7(C24G) were transiently transfected to express GFP-NHA9 and GFP-N98 and stained for immunofluorescence microscopy with the indicated antibodies. (**A**) Expression of GFP-NHA9 (green) provoked a reduced LAP2α staining (magenta) in the nucleoplasm in E7(C24G) cells, (**B**) but not in cells lacking RB due to the expression of wild-type E7. Expression of GFP-N98 (green) had no impact on the LAP2α pattern in either background. (**C**) Lamin A/C (LA/C, magenta) was displaced to the nucleoplasm in the presence of GFP-NHA9 (green) and adopted a punctuated pattern in E7(C24G) cells as compared to E7 expressing cells (**D**). In contrast, LA/C was enriched at the nuclear envelope (NE) in GFP-N98 (green) expressing cells, irrespective of the RB status. Shown are representative confocal images. Scale bars, 5 μm. (**E**) Fluorescence intensity of LAP2α and LA/C staining was determined along the axis shown as line in the fluorescence images and plotted as a graph. Fluorescence intensity was normalized to DAPI. (**F**) Quantification of cells with altered LAP2α distribution. About 400 cells were analyzed for each sample. The number of experiments for LAP2α and LA/C staining was: *n*  =  4; * *p* <  0.05, ns, *p* not significant, *t*-test, two-tailed.

Next, we transiently expressed GFP-NHA9 and GFP-N98 in these stable cell lines. As shown in Figure 2A,B, the fluorescence signal for LAP2α was largely decreased upon expression of GFP-NHA9 in E7(C24G) cells, i.e., in the presence of RB (Figure 2A), but not in E7 expressing cells, i.e., in the absence of the pocket proteins (Figure 2B). Similarly, expression of GFP-NHA9 in E7(C24G) cells provoked perturbation in LA/C localization in the nuclear lamina and the nucleoplasm (Figure 2C), which was less pronounced in cells lacking the pocket proteins due to E7 expression (Figure 2D). Respective fluorescence intensity profiles for LAP2α and LA/C staining after normalization to DAPI are shown in Figure 2E. About 70% of GFP-NHA9 expressing cells presented with deformed nuclei in E7(C24G) cells but only about 25% in E7 expressing cells (Figure 2F). GFP-N98 expression had no impact on LAP2α localization and accessibility both in the E7(C24G) and E7 backgrounds. Likewise, LA/C localization to the nuclear lamina was indistinguishable (Figure 2C,D). In both backgrounds, 20–25% of the cells exhibited deformed nuclei (Figure 2F). Together our data suggest that NHA9 expression in cells lacking RB circumvented NHA9-provoked NE morphology changes, either in TKO MEFs or by E7-driven RB degradation.

### 3.3. Epigenetic Dysregulation Correlating with NHA9 Expression

**Figure 3:**
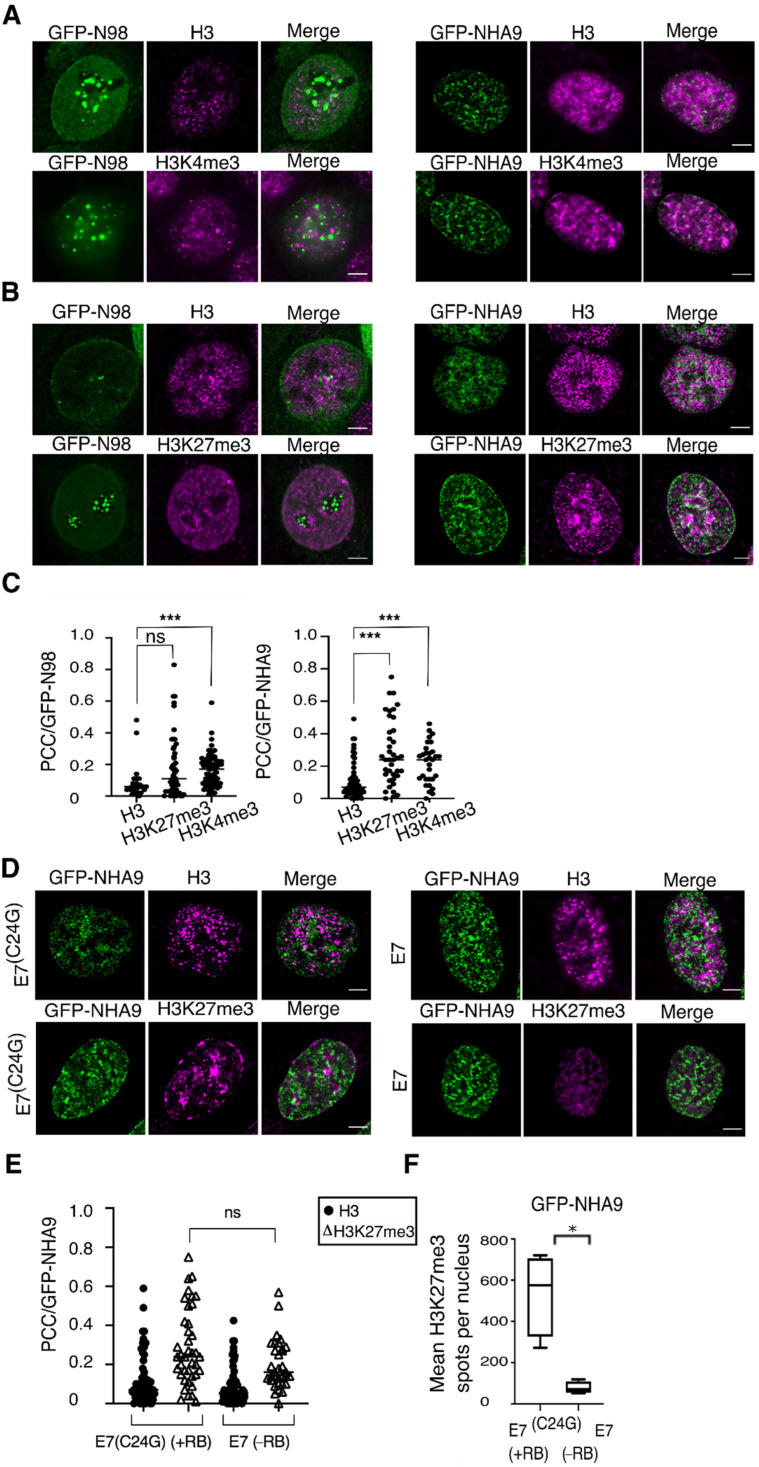
NUP98–HOXA9 foci associate with H3K4me3, and H3K27me3 marks and alters H3K27me3 distribution. (**A**) HCT-116 cells transiently expressing GFP-NHA9 (green) and GFP-N98 (green) were respectively stained for histone H3 (magenta) and the H3K4me3 mark (magenta), as well as (**B**) for H3 (magenta) and H3K27me3 (magenta). Shown are representative confocal images. Scale bars, 5 μm. (**C**) Pearson’s correlation coefficients (PCCs) GFP-NHA9 as well as GFP-N98 with H3K27me3 and H3K4me3. Histone 3 was used for normalization. (**D**) The effect of GFP-NHA9 on H3K27me3 patterning is neglected in cells expressing E7 and lacking RB but not in E7(C24G) expressing cells. The pattern of H3 is similar in both backgrounds. (**E**) PCC values regarding GFP-NHA9 with H3K27me3 and H3 in E7(C24G) and E7 expressing cells. (**F**) Quantitative measurement of H3K27me3 spots per nucleus in E7(C24G) and E7 cells expressing GFP-NHA9. The number of experiments was: *n*  =  3; *** *p* <  0.001, * *p* <  0.05, ns, *p* not significant, *t*-test, two-tailed.

Given the architectural effects of NHA9 on nuclear organization, we next asked whether these changes provoked by NHA9 translate into changes in chromatin structure, as not only are LAP2α and LA/C known to be involved in the regulation of chromatin [37,38,39], but also RB itself has important chromatin regulatory functions [37,38,54]. As a read-out for the analysis of chromatin structure, we decided to analyze the pattern of some histone modifications, known to be correlated to N98, NHA9, RB, and/or LAP2α, i.e., trimethylation of histone H3K4, H3K27, as well as H3K9 [4,12,41,42,43,44,45,46,47,48]. To explore the impact of NHA9 expression on histone methylation in an RB-dependent manner, we transiently expressed GFP-NHA9 in HCT-116 cells and proceeded with immunofluorescence analysis of H3K27me3 and H3K4me3 deposition under these conditions. Representative images of the respective staining patterns are shown in Figure 3A,B. For a quantitative assessment of the respective relationship between GFP-NHA9 and the histone marks, we employed the Pearson Correlation Coefficient (PCC) analysis [55]. This quantitative method emphasizes the degree of colocalization between two fluorophores. Histone H3 distribution was used for normalization and the correlation between GFP-N98 and H3K4me3 as method control, given the known interaction between N98 and MLL [4,12,41]. Our analysis revealed a low correlation of GFP-NHA9 as well as GFP-N98 with histone H3 (PCC = 0.07 and 0.06, respectively), but a significantly increased correlation between GFP-NHA9 and H3K4me3 (PCC = 0.24, *p* < 0.001; Figure 3A,C), as well as GFP-N98 and H3K4me3 (PCC = 0.17, *p* < 0.001; Figure 3A,C). When examining H3K27me3, we found that GFP-NHA9 expression caused an enrichment of the H3K27me3 mark at the nuclear periphery and in high-density clusters in the nuclear interior, in highly condensed chromatin regions, as opposed to the more uniformly punctate pattern seen in GFP-N98 expressing cells. The correlation of GFP-NHA9 and H3K27me3 was significantly higher as compared to H3 (PCC = 0.25, *p* <  0.001; Figure 3B,C), while the correlation between GFP-N98 and H3K27me3 (PCC = 0.11, *p* < 0.001) was not significantly higher as compared to total H3 (Figure 3C). Additionally, GFP-NHA9 correlated with H3K9me3 (PCC = 0.20, *p* < 0.001), similar to values recently described [56] but without perturbing the overall H3K9me3 pattern (Figure S2A). Together these data indicate that NHA9 localization in the nucleus correlates with distinct histone methylation marks, but it only perturbs H3K27me3 distribution.

As outlined above, RB facilitates heterochromatin formation via EZH2-mediated deposition of H3K27me3 [45,46,47]. Given the apparent link between RB and NHA9 provoked changes in nuclear architecture (Figure 1 and Figure 2) and the impact of NHA9 expression on H3K27me3, we next visualized the spatial distribution of the H3K27me3 mark in response to the NHA9 expression as a function of RB. We examined the E7(C24G) and E7 expressing HCT-116 cell lines. The images revealed that, in GFP-NHA9 expressing E7(C24G) cells, H3K27me3 was enriched at the nuclear periphery and formed compartmentalized regions in the nucleus (Figure 3D,F, E7(C24G)), as described above (Figure 3B). In the absence of RB due to the expression of E7, H3K27me3 remained uniformly distributed throughout the nucleoplasm in the typical punctate pattern despite the presence of GFP-NHA9 (Figure 3D and Figure S2B). The colocalization studies did not indicate a significant alteration in the PCC values between GFP-NHA9 and H3K27me3 regardless of the presence or absence of RB (Figure 3E and Figure S2C). The same was true for the distribution pattern of H3 (Figure 3E). However, the number of H3K27me3 foci in E7(C24G) cells expressing GFP-NHA9 appeared six times higher than in E7 cells under the same conditions (Figure 3F and Figure S2C). Furthermore, H3K27me3 intensity profiles across the nuclear diameter confirmed the presence of peaks throughout the nucleoplasm in E7(C24G) cells (Figure S2D, E7(C24G)). In contrast, although NHA9 nuclear foci were frequently associated with H3K4me3 (Figure 3C), the intranuclear distribution of those nuclear foci was similar in E7(C24G) cells and E7 cells (Figure S2E). These results indicate that H3K27me3 foci are not casually positioned in the nucleus in cells expressing NHA9 but are associated with RB.

### 3.4. Association of Polycomb-Group Proteins with NHA9 Appears to Depend on RB

**Figure 4:**
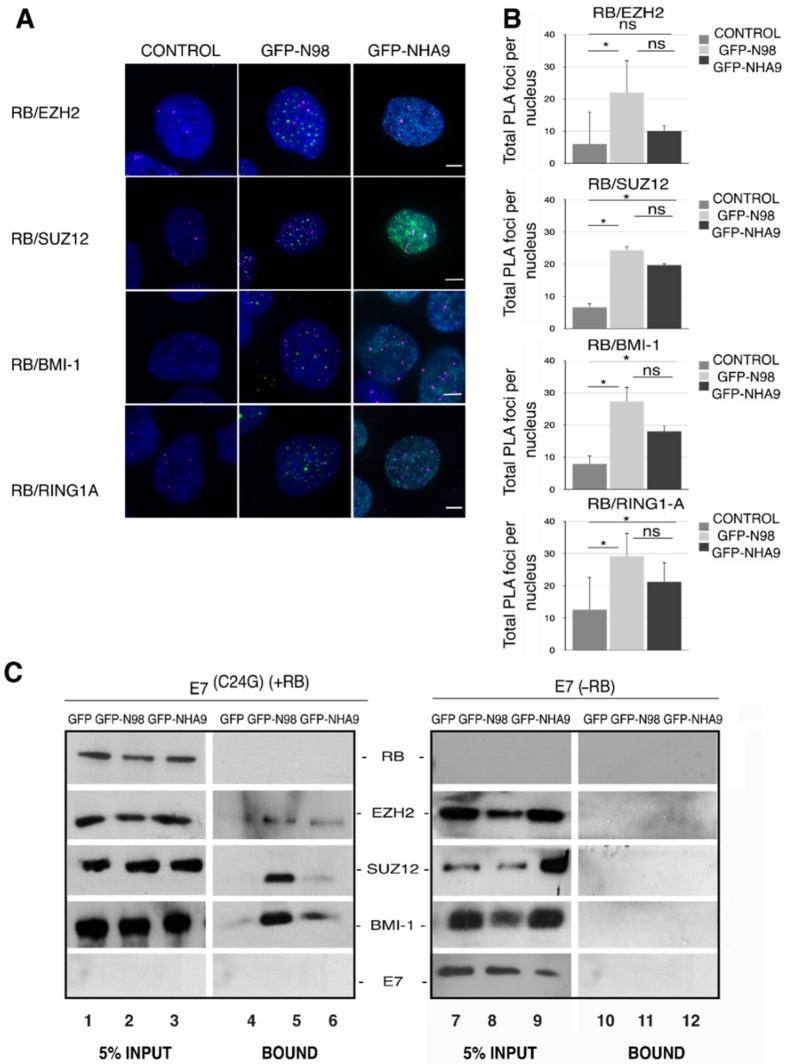
Association of RB with PcG proteins in NUP98–HOXA9 expressing cells. Respective proximity ligation assays (PLA) between RB and PcG proteins EZH2, SUZ12, BMI-1, and RING1A in HCT-116 cells expressing GFP-NHA9 (green) or GFP-N98 (green). Representative immunofluorescence images are shown in (**A**) DAPI (blue) staining was used to visualize the nuclei. Scale bars, 5 μm. (**B**) PLA foci per cell (magenta) were quantified using Fiji/ImageJ. About 400 cells were analyzed for each sample; the number of experiments was: *n*  =  3. * *p* <0.05, ns, *p* not significant, *t*-test, two-tailed. (**C**) GFP-trap assays to study the potential interaction of NHA9 with PcG proteins EZH2, SUZ12, and BMI-1. GFP-NHA9 were transiently expressed in HCT-116 cells; GFP and GFP-N98 expression was employed as control. After 48 h, GFP proteins and associated factors were recovered from cell lysates and probed by Western blot analysis using antibodies against EZH2, SUZ12, and BMI-1. The proteins had the following expected molecular weight: RB:110 kDa; EZH2: 98 kDa; SUZ12: 83 kDa; BMI1: 41 kDa; E7: 20 kDa. The number of experiments was: *n*  =  3.

Having seen the RB-dependent alterations in H3K27me3 deposition in cells expressing NHA9, we next analyzed chromatin remodeling proteins directly involved in H3K27me3 catalysis. H3K27me3 is regulated by the Polycomb-group of proteins (PcG), which comprise polycomb repressive complexes 1 and 2 (PRC1 and PRC2). H3K27me3 placed by PRC2 is recognized by PRC1 complexes [57,58,59]. In order to explore the engagement between polycomb complexes and RB in NHA9 expressing cells, we carried out proximity ligation assays (PLA; [60]) in HCT-116 cells expressing GFP-NHA9. We analyzed the proximity between RB and the two PRC2 proteins EZH2 and SUZ12 (suppressor of zeste 12) on the one hand, as well as of RB and the PRC1 members BMI-1 (B cell-specific Moloney murine leukemia virus integration site 1) and RING1A. As shown in Figure 4A,B, 5–10 PLA foci/cell were detected for RB with any of the four PcG proteins in untransfected control cells. The number of PLA foci increased significantly in GFP-NHA9 expressing cells, except for RB and EZH2 (Figure 4A,B), and similarly upon expression of GFP-N98. The positive and negative controls for the PLA assays are shown in Figure S3A. Together our data suggest that NHA9 and N98 somewhat augment the association of RB with endogenous PcG proteins.

To further support this notion, we next performed GFP trap affinity purification assays in combination with Western blot analysis of lysates from HCT-116 cell lines stably expressing E7 and E7(C24G), respectively. GFP-NHA9 and GFP-N98, in contrast to GFP alone, both copurified EZH2, SUZ12, and BMI-1 (the RING1A antibody did not perform in Western blot) in the presence of RB (Figure 4C, lanes 4–6) but not in its absence (Figure 4C, lanes 11 and 12). These data indicate that not only RB proteins but also GFP-NHA9 and GFP-N98 associate with PcG proteins. Association of NHA9 (and N98) with PcG proteins necessitates the presence of RB. In line with the PLA assays, copurification of SUZ12 and BMI-1 was reduced in GFP-NHA9 as compared to GFP-N98 expressing cells (Figure 4C, lanes 5 and 6). Successful transfections and expression of the proteins were confirmed by probing with antibodies against GFP and N98 (Figure S3B).

## 4. Discussion

In a previous study, we described in detail an aberrant nuclear envelope phenotype caused by the expression of NUP98 fusion proteins in transfected human cell lines, in transduced mouse bone marrow cells, and in patient-derived samples [23]. We showed here that for NHA9, the appearance of these morphological alterations was largely diminished in the absence of RB, both in human cells expressing the HPV 16 E7 protein and in TKO MEFs. Furthermore, our data suggested that the aberrant NE phenotype correlated with changes in chromatin organization, in particular trimethylation of histone H3K27, in an RB-dependent manner.

N98 fusion proteins and N98 are known to interact with DNA and chromatin and to play regulatory roles in gene expression at distinct levels [14,20,22,24,26,27,41,42,61,62,63,64,65]. Our study confirmed the bivalent association of NHA9 with the active histone modification mark H3K4me3, as well as the facultative heterochromatin mark H3K27me3 ((Figure 3); see also [65]). Moreover, NHA9 not only correlated with H3K27me3, but also caused an enrichment of the mark at the nuclear periphery and in high-density clusters in highly condensed chromatin regions (Figure 3). Consistent with these chromatin rearrangements, a chromosome conformation analysis using Hi-C revealed that NHA9 induced the organization of an aberrant 3D chromatin structure during cellular transformation that was enriched in proto-oncogenes [56]. Our data further strengthened the notion that N98 fusion proteins and N98 bind to H3K4me3 residues, likely due to the interaction with MLL [41,42,43,44,61,66].

Our study revealed that depletion of RB prevented (i) a chromatin reorganization provoked by NHA9 expression and (ii) largely reduced the correlation between NHA9 and H3K27me3. H3K27me3 was enriched in heterochromatin clusters in the presence of NHA9 (Figure 4) but not in control cells. What could be the role of RB in NHA9-provoked changes in nuclear architecture and leukemia? RB is a nuclear and chromatin-associated protein with a multitude of molecular functions that go beyond transcriptional control of cell cycle genes, such as regulation of autophagy, apoptosis, and stemness [30,31]. As for transcriptional control of cell cycle genes, those functions are linked to CDK-dependent E2F target gene transcription. A second pool of RB appeared to impact heterochromatin organization and genome stability in a CDK- and phosphorylation independent manner. In this context, it has been shown that RB directed H3K27me3 via EZH2. This has been shown, for example, for transcriptional enhancers and promoters of the pluripotency genes *OCT4* and *SOX2* and for repetitive DNA sequences [46,47,67]. Our data presented here suggested that NHA9 expression perturbed H3K27me3 deposition in an RB-dependent manner.

The activation of *HOX* gene clusters, in particular, posterior *HOXA* genes, by N98 fusion proteins is well-established. The persistent expression of the posterior *HOXA* cluster, often in particular *HOXA9,* in N98-related leukemia is thought to be a major cause for the arrested terminal differentiation of myeloid precursor cells (for review see: [68,69]). *HOX* gene expression is, amongst others, regulated by RB [70]. *HOX* genes are organized as facultative heterochromatin [71]. Given the role of RB in heterochromatin organization, it is conceivable that RB might be important for *HOX* organization as facultative heterochromatin and, hence, *HOX* expression. Therefore, it will be interesting to determine the impact of RB depletion on the expression of NHA9 target genes.

Silencing of *HOX* genes is typically achieved by polycomb-spreading [72]. RB associates with PcGs (Figure 4), as previously described [73], and regulates expression of EZH2 [46,74,75], underlining the strong link between *HOX* gene expression, polycomb regulation, and RB. H3K27me3 is placed by the PRC2 complex and recognized by the PRC1 complex. Our PLA and GFP-trap assays revealed that not only RB but also NHA9 and N98 associate with PcG members (Figure 4), and that both enforce the association between RB and PcG proteins. This may affect the maintenance of some PcG-dependent repressed transcriptional states, for example of *HOXA* clustered genes. Importantly, the association of NHA9 (and N98) with PcG complexes is interrupted in cells lacking RB, suggesting that NE alteration caused by NHA9 expression is linked to RB-dependent PcG-mediated H3K27me3 rearrangements. Together, our data suggest that RB might be a key regulator of *HOXA* gene activation in N98-related leukemia, and that NHA9 may interfere with proper RB-dependent silencing of *HOXA* clustered genes by PcG proteins. Future studies are necessary to address this question.

Another hallmark of N98-related leukemia is the poor prognosis for patients due to moderate response to DNA-damaging agents used in chemotherapy [76]. RB is required for functional non-homologous end joining (NHEJ) and homologous-recombination based repair of DNA double-strand breaks (DSBs; [77,78]). RB loss renders cells more sensitive to DNA-damaging agents [79,80]. N98 fusion proteins have been shown to alter DSB repair, in particular NHEJ ([81]; our unpublished data). It is therefore tempting to speculate that RB loss/inactivation would improve chemotherapy outcome in patients harboring N98 chromosomal translocations.

## Supplementary Materials

The following figures are available online at https://www.mdpi.com/article/10.3390/cells10112851/s1, Figure S1: NE phenotype alterations mediated by NHA9; Figure S2: Histone patterns and colocalization coefficients; Figure S3: PLA Technical controls.
