# Supplementary material for: The Retinoblastoma Tumor Suppressor Is Required for the NUP98-HOXA9-Induced Aberrant Nuclear Envelope Phenotype
Source: Cells. 2021 Oct 22;10(11):2851. doi: 10.3390/cells10112851 (PMC8616146; doi:10.3390/cells10112851)
Supplement: Supplementary file 1 [file cells-10-02851-s001.zip › cells-1407607-supplementary.pdf]

Supplementary Materials

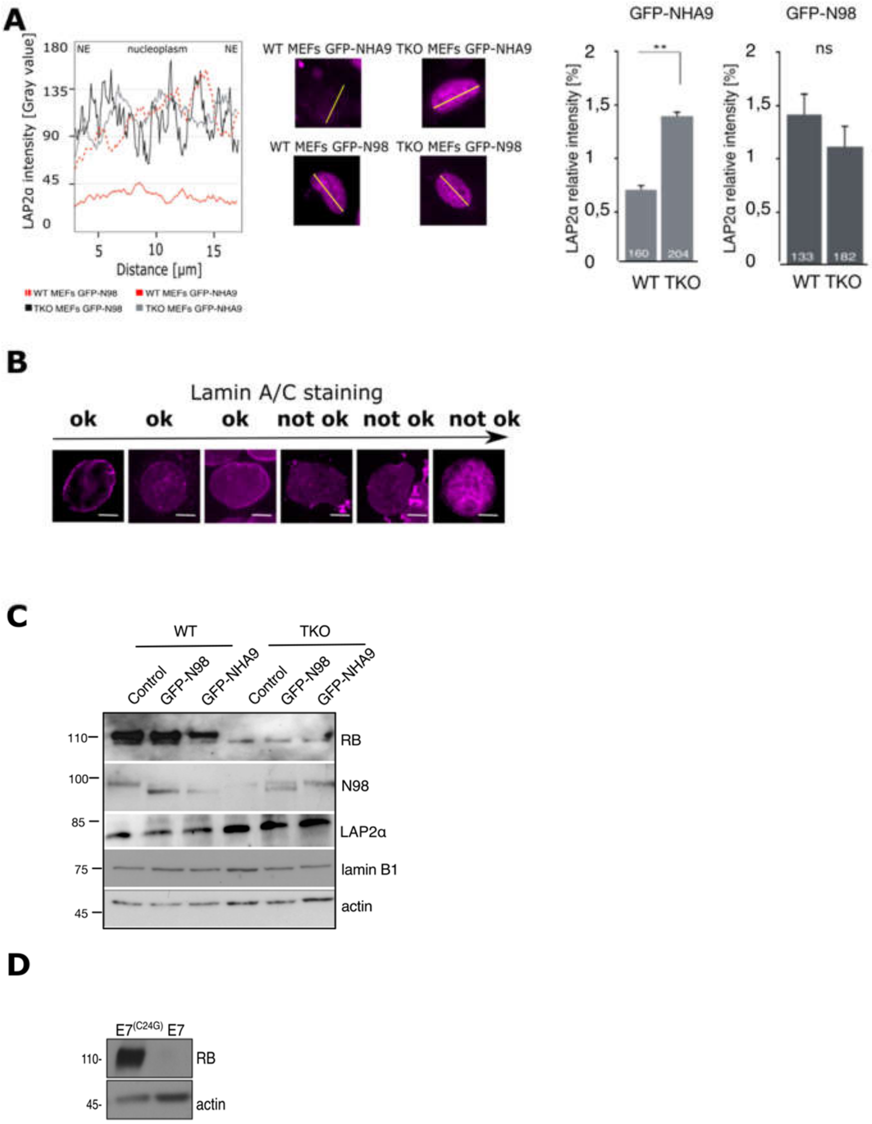

Figure S1. NE phenotype alterations mediated by NHA9. (**A**) Fluorescence intensity of LAP2α was determined along the axis shown as line in the fluorescence images and plotted as graph. Quantification of the fluorescence intensity of LAP2α staining by normalization to DAPI (4′,6-diamidino-2-phenylindole; not shown). (**B**) Changes in lamin A/C morphology. Scale bars, 5 µm. (**C**) Western blot analysis of the expression levels of RB, N98, LAP2α, and LB1 in MEFs and TKO cells. Actin was used as loading control. The number of experiments was: $n = 4$. (**D**) Western blot using antibody against retinoblastoma protein in E7(C24G) and E7 expressing cells. Actin was used as loading control.

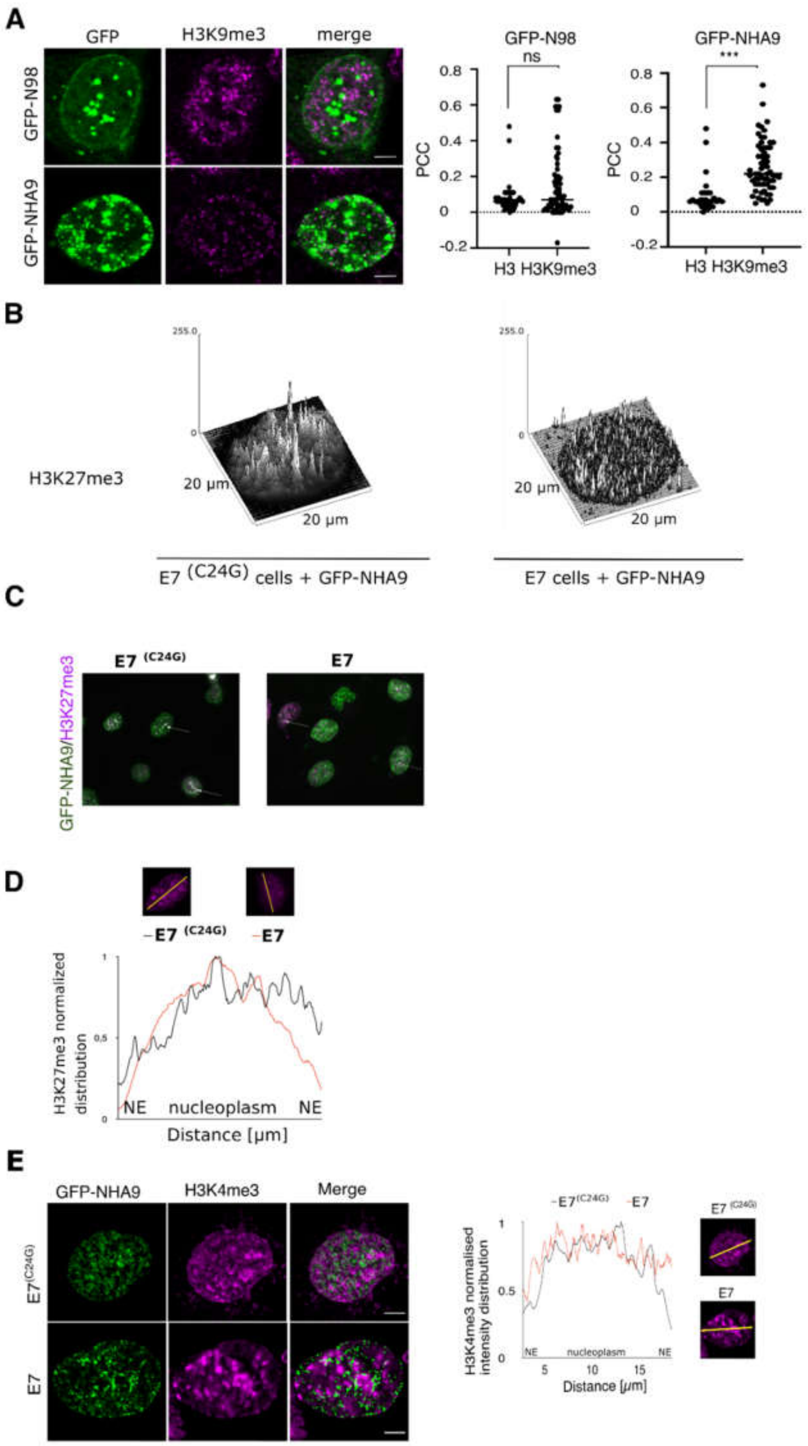

Figure S2. Histone patterns and colocalization coefficients. (**A**) Immunofluorescence labeling and correlation analysis for GFP-N98 and GFP-NHA9 (green) and H3K9me3 (magenta). Shown are representative confocal images. Scale bars, 5 µm. Pearson's correlation coefficients (PCCs) for GFP-N98 and GFP-NHA9 with H3K9me3 were calculated using histone 3 as the control. (**B**) Overall pattern of H3K27me3 distribution shown as surface plot along an axis and as line in the

fluorescence images. H3K27me3 changes on E7 and E7(C24G) cells expressing GFP-NHA9 visualized on 3D plots. Cells were fixed and stained with H3K27me3 antibodies. (**C**) Colocalization (white) of GFP-NHA9 (green) with H3K27me3 (magenta) in E7 (C24G) and E7 expressing cells. Scale bars, 10 µm. (**D**) Pattern of the overall distribution of H3K27me3 on E7 or E7(C24G) cells expressing GFP-NHA9 shown as surface plot along an axis as line in the fluorescence images. (**E**) Representative wide-field images of H3K4me3 in cells expressing GFP-NHA9 and E7 (C24G) or E7. Scale bars, 5 µm. Pattern of the overall distribution of H3K4me3 shown as surface plot along an axis and as line in the fluorescence images.

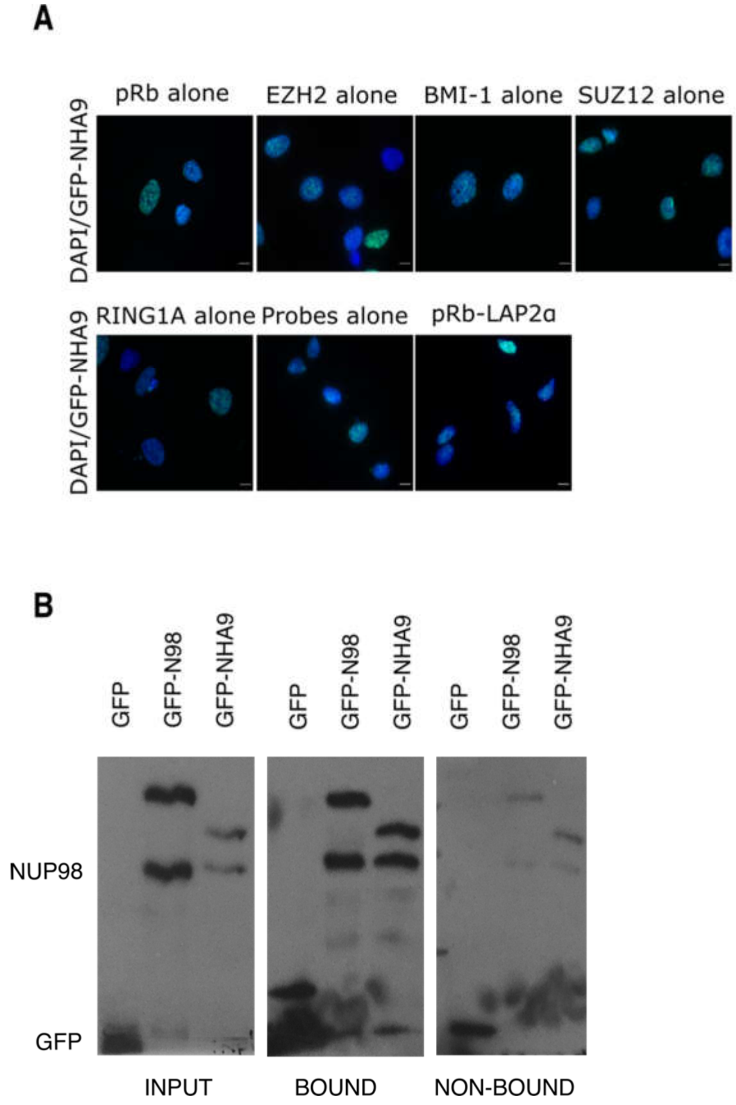

Figure S3. PLA Technical controls. (**A**) Technical controls for proximity ligation assay in cells expressing GFP-NHA9. No PLA signal was generated when RB, EZH2, BMI1, SUZ12, and RING1A antibodies were applied alone, as with the probes alone. The combination of anti-RB and anti-LAP2α antibodies was used as a positive control. Duolink® PLA was then performed using anti-rabbit PLUS and anti-mouse MINUS PLA probes. DAPI staining was used to visualize the nuclei, magenta (PLA signals). Images are representative from at least 3 independent experiments. Scale bars, 10 µm. (**B**) Successful transfection and expression of the proteins was confirmed by probing with antibodies against GFP and N98. The number of experiments was: $n = 3$.
